# Real-World Experience of Chronic Hepatitis C-Related Compensated Liver Cirrhosis Treated with Glecaprevir/Pibrentasvir: A Multicenter Retrospective Study

**DOI:** 10.3390/jcm10225236

**Published:** 2021-11-10

**Authors:** Pei-Yuan Su, Yang-Yuan Chen, Jun-Hung Lai, Hung-Ming Chen, Chih-Ta Yao, I-Ling Liu, Ya-Huei Zeng, Siou-Ping Huang, Yu-Chun Hsu, Shun-Sheng Wu, Fu-Yuan Siao, Hsu-Heng Yen

**Affiliations:** 1 Division of Gastroenterology, Department of Internal Medicine, Changhua Christian Hospital, Changhua 500, Taiwan; 111252@cch.org.tw (P.-Y.S.); 27716@cch.org.tw (Y.-Y.C.); 125267@cch.org.tw (I.-L.L.); 120693@cch.org.tw (Y.-H.Z.); 182972@cch.org.tw (S.-P.H.); 77149@cch.org.tw (Y.-C.H.); 18840@cch.org.tw (S.-S.W.); 2 Division of Gastroenterology, Department of Internal Medicine, Yuanlin Christian Hospital, Changhua 500, Taiwan; 3 Department of Hospitality, MingDao University, Changhua 500, Taiwan; 4 Division of Gastroenterology, Department of Internal Medicine, Erhlin Christian Hospital, Changhua 500, Taiwan; 61004@cch.org.tw; 5 Division of Gastroenterology, Department of Internal Medicine, Yunlin Christian Hospital, Yunlin 648, Taiwan; 820199@cch.org.tw; 6 Division of Gastroenterology, Department of Internal Medicine, Lukang Christian Hospital, Changhua 500, Taiwan; 704150@cch.org.tw; 7 Department of Emergency Medicine, Changhua Christian Hospital, Changhua 500, Taiwan; 57385@cch.org.tw; 8 Department of Kinesiology, Health and Leisure, Chienkuo Technology University, Changhua 500, Taiwan; 9 Artificial Intelligence Development Center, Changhua Christian Hospital, Changhua 500, Taiwan; 10 General Education Center, Chienkuo Technology University, Changhua 500, Taiwan; 11 Department of Electrical Engineering, Chung Yuan University, Taoyuan 320, Taiwan; 12 College of Medicine, National Chung Hsing University, Taichung 400, Taiwan

**Keywords:** hepatitis C, therapy, cirrhosis, compensated cirrhosis

## Abstract

Background: Glecaprevir/pibrentasvir is a protease inhibitor-containing pangenotypic direct-acting antiviral regimen that has been approved for the treatment of chronic hepatitis C. The present study aimed to evaluate the safety and efficacy of glecaprevir/pibrentasvir in patients with compensated cirrhosis in a real-world setting. Methods: We evaluated the real-world safety and efficacy of glecaprevir/pibrentasvir in patients with compensated cirrhosis from five hospitals in the Changhua Christian Care System, who underwent treatment between August 2018 and October 2020. The primary endpoint was a sustained virological response observed 12 weeks after completion of the treatment. Results: Ninety patients, including 70 patients who received the 12-week therapy and 20 patients who received the 8-week therapy, were enrolled. The mean age of the patients was 65 years, and 57.8% of the patients were males. Sixteen (17.8%) patients had end-stage renal disease, and 15 (16.7%) had co-existing hepatoma. The hepatitis C virus genotypes 1 (40%) and 2 (35.6%) were most common. The common side effects included anorexia (12.2%), pruritus (7.8%), abdominal discomfort (7.8%), and malaise (7.8%). Laboratory adverse grade ≥3 events included anemia (6.3%), thrombocytopenia (5.1%), and jaundice (2.2%). The overall sustained virological response rates were 94.4% and 97.7% in the intention-to-treat and per-protocol analyses, respectively. Conclusions: the glecaprevir/pibrentasvir treatment regimen was highly effective and well tolerated among patients with compensated cirrhosis in the real-world setting.

## 1. Introduction

Chronic hepatitis C virus (HCV) infection is a common cause of liver cirrhosis and liver cancer, resulting in significant morbidity and mortality [1,2,3]. Approximately 71 million individuals have been estimated to be chronically infected worldwide, accounting for 1% of the world’s population, with 10–20% of infected individuals developing complications over 20–30 years [4]. Over the past two decades, interferon-based therapy has been the standard treatment for this condition, but it is limited by its side effects, especially among individuals with cirrhosis [2,5]. However, HCV treatment has been revolutionized recently by the introduction of direct-acting antiviral (DAA) therapy, which has a high treatment success rate and safety profile. The Taiwanese health care system has been reimbursing these new agents since 2017, and the government has set the goal of obtaining an 80% treatment coverage rate with DAAs by 2025 [1,6,7,8,9,10]. Nevertheless, the treatment of patients with compensated cirrhosis is challenging in the early developmental stage of DAAs.

Glecaprevir/pibrentasvir (GLE/PIB) is an interferon- and ribavirin-free, pangenotypic DAA approved for the treatment of chronic HCV infection in patients with or without compensated cirrhosis [7,11]. It is also the first pan-genotype DAA approved for patients with renal dysfunction [12]. Since August 2018, GLE/PIB has been reimbursed in Taiwan for patients with confirmed HCV viremia and no prior DAA therapy [13]. The EXPEDITION-1 study, involving patients with compensated cirrhosis, demonstrated a sustained virologic response (SVR)12 rate of 99% after the 12-week therapy [14], and the subsequent EXPEDITION-8 study achieved an SVR12 rate of 98.0% with the shortened 8-week therapy for compensated cirrhosis [11]. No patients prematurely discontinued treatment owing to any adverse events in the trial setting. Until now, data on the efficacy and safety of GLE/PIB in patients with compensated cirrhosis are still limited in the real-world setting [15,16], especially for the Asian population. Thus, this study aimed to evaluate the real-world safety and efficacy of GLE/PIB in patients with compensated cirrhosis from five hospitals in the Changhua Christian Care System [9,17].

## 2. Materials and Methods

### 2.1. Materials

This retrospective study included DAA treatment-naïve patients with compensated liver cirrhosis, defined as having a Child–Pugh score of 5 or 6, undergoing treatment for HCV infection, who received ≥1 dose of GLE/PIB between August 2018 and October 2020 at five hospitals of the Changhua Christian Health Care System. The study was approved by the Changhua Christian Hospital Institutional Review Board (CCH IRB No. 190814), and the requirement for informed consent was waived because the study was conducted retrospectively. Medical information, including demographics, baseline medical conditions, anti-HCV treatment regimen and duration, laboratory analysis, and information on adverse events, was extracted from electronic patient records. A diagnosis of cirrhosis was made by one of the following findings: (a) liver biopsy with a meta-analysis of histological data in viral hepatitis fibrosis score of 4 (or equivalent); (b) documented evidence of liver cirrhosis with transient elastography (≥F4 by Fibroscan (Echosens France); (c) FIB-4 ≥ 3.25 and aspartate aminotransferase (AST)-to-platelet ratio index (APRI) of >2 [18]; (d) sonographic evidence of liver cirrhosis and portal hypertension [11,19]. The anti-HCV treatment response at the end of treatment was compared with that 12 weeks after the treatment. All procedures were conducted in accordance with relevant guidelines and regulations of the National Health Insurance Administration of Taiwan.

### 2.2. Treatment, Efficacy and Safety Evaluation

Our primary goal was to evaluate the treatment results of GLE/PIB in patients with compensated cirrhosis. We used ART HCV assays (real-time HCV and HCV genotype II, Abbott Molecular, Abbott Park, IL, USA) to quantify HCV RNA concentrations and genotyping. The end-of-treatment viral response (ETVR) was defined as an HCV RNA level lower than the lower limit of quantification (LLOQ) upon completion of the treatment course. An SVR was defined as an HCV RNA level lower than the LLOQ at 12 weeks after the last medication. The treatment period was 12 and 8 weeks for patients with compensated liver cirrhosis before and after April 2020, according to the drug label approved by Taiwan Food and Drug Administration. Virologic treatment failure was defined as either (a) non-response (HCV was detectable during and at the end of treatment); (b) relapse (HCV was undetectable at the end of treatment, but detectable during the follow-up period). The following two endpoints for viral response were evaluated: ETVR at the end of therapy and SVR after 12 weeks of antiviral therapy. The intention-to-treat group (ITT) included patients who received at least one dose of GLE/PIB, and the per-protocol group (PP) was established by excluding patients because of non-virologic failure.

### 2.3. Statistical Analyses

Statistical analyses were performed using the PS IMAGO Pro 7 software and MedCalc statistical software, version 19.8 (2021 MedCalc Software Ltd., Ostend, Belgium). Baseline data were analyzed to compare the two course groups (8 weeks/≥12 weeks) using either Student’s *t*-test or the Mann–Whitney U test for continuous data, and the chi-square test or Fisher’s exact test for categorical data. The distribution of continuous variables was checked using the one-sample Kolmogorov–Smirnov test. The Wilcoxon signed-rank test or the paired *t*-test was used to compare continuous variables between baseline and SVR12. The results were considered statistically significant if the two-tailed *p*-value was <0.05 for all tests.

## 3. Results

### 3.1. General Characteristics of the Study Population

**Figure 1:**
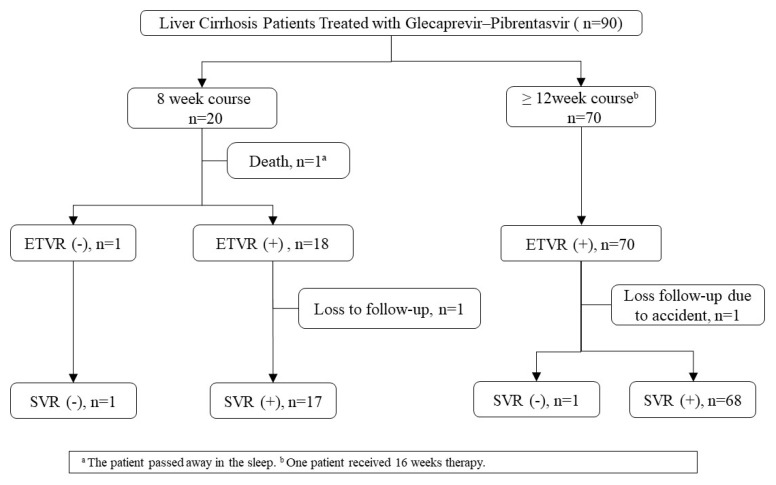
Flowchart showing baseline data of hepatitis C virus compensated cirrhotic patients with glecaprevir/pibrentasvir.

A total of 90 patients, with compensated cirrhosis and HCV infection, received GLE/PIB-based anti-HCV therapy during the study period (Figure 1 and Table 1). Twenty patients underwent an 8-week treatment course, while the remaining underwent a 12-week treatment course. Most patients were male (57.8%), with a mean age of 65 years. Approximately 17.8% of the patients had end-stage renal disease. A higher proportion of patients in the 8-week group had hepatoma (35% vs. 11.4%, *p* = 0.036). The most common HCV genotype observed was type 1 (40%), followed by type 2 (35.6%), type 3 (13.3%), type 6 (3.3%), and others (7.8%). One fatality was observed during the treatment period, which was unrelated to the treatment. Two patients were lost to follow-up after achieving an end-of-treatment response. The overall ITT SVR rate was 94.4%, and the PP SVR rate was 97.7% (Table 2).

### 3.2. The Safety Profile of the Treatment

Anorexia was the most reported side effect of GLE/PIB treatment, followed by pruritus, malaise, and abdominal discomfort (Table 3). A significant increase (≥5× elevation) in the levels of bilirubin, glutamic oxaloacetic transaminase (GOT), and glutamic pyruvic transaminase (GPT) was observed in 2.2% of the study population. Grade 3 anemia was observed in 6.3% of the patients, and grade 3 thrombocytopenia was observed in 5.1% of the patients. There were no side effects related to premature treatment termination in the study population.

### 3.3. Laboratory Change Achieving SVR12

Table 4 summarizes the change in laboratory parameters among the patients who achieved SVR12. Significant improvements in liver function, including GOT, GPT, albumin, bilirubin, and prothrombin time, were observed after treatment. The FIB-4 level improved from 4.49 to 2.86 (*p* < 0.001), and the APRI index improved from 1.309 to 0.504 (*p* < 0.001) after SVR12. The model of end-stage liver disease scores also improved from 6.905 to 5.47 (*p* = 0.012). There were no changes in platelet count, hemoglobin level, and creatinine level after treatment.

## 4. Discussion

In this multicenter cohort study of patients with compensated liver cirrhosis and HCV, who received GLE/PIB therapy, the overall SVR rate was 94.4%. Despite the higher proportion of comorbidities (such as renal failure or hepatoma), our results are comparable to those observed in the EXPEDITION-1 and -8 trials [11,14], or other real-world reports [16,19,20], indicating that GLE/PIB is a highly effective treatment regimen for HCV infection in the real world.

GLE/PIB has been approved as an interferon-free pangenotypic DAA for hepatitis C patients with or without compensated cirrhosis and renal dysfunction [7,11,21]. In a pooled analysis of 2369 patient data from nine phase 2 and 3 clinical trials, with 13% of the patients having compensated cirrhosis, the overall SVR12 rates were 96.4% with and 97.5% without cirrhosis [20]. This trial setting included only 6% of the Asian population; hepatoma was present in 2% and renal dysfunction was present in 5% of the study population. Despite the high SVR rate in the clinical trial setting, patients with unfavorable factors, such as advanced age [21], comorbidities [22], or concurrent malignancy [23], were commonly excluded, and the data collected may overestimate the performance of the investigated regimen. Real-world experience is important to further generalize the trial data to real-world populations. The experience of GLE/PIB in the real-world report is lacking for those with compensated cirrhosis, especially for the Asian population. In one meta-analysis, involving 12,531 adults treated with G/P from 18 real-world reports [15], only 362 were cirrhotic. The recent German Hepatitis C-Registry report [24] was the largest to date, reporting the effectiveness and safety of 187 patients with compensated cirrhosis, who received the 8-week GLE/PIB therapy. The SVR was 98.4% (127/129) in the PP analysis and 85.8% (127/148) in the ITT analysis. No adverse events resulting in drug discontinuation occurred. One strength of the present study was that we included patients from the Changhua Health Care System, including one medical center and four local hospitals. The present study provided further evidence of the efficacy of GLE/PIB for patients with compensatory liver cirrhosis, who have a high SVR rate of 94.4% in the real-world setting.

GLE/PIB was first approved for a 12-week treatment course in patients with compensated cirrhosis, based on the EXPEDITION-1 trial [14] in 2017 and the SURVEYOR-II [25] trial in 2018 for genotype 3, with an SVR rate of >96%. The subsequent EXPEDITION-8 trial further demonstrated that the simplified treatment course maintained a high SVR rate of 98% in the case of treatment-naïve patients with compensatory liver cirrhosis. Such a shortened treatment, without compromising the therapeutic effect, is appealing to improve patient compliance, especially in the era of the coronavirus disease pandemic. Our analysis found that the 8-week course had an SVR12 rate similar to that of the 12-week course, despite the higher proportion of patients with hepatocellular carcinoma (HCC) and the higher APRI score in the 8-week group. Only one patient in the 8-week group (genotype 1) and another in the 12-week group (genotype 3) experienced virologic failure in the present study. The common treatment-related side effects included anorexia, malaise, abdominal discomfort, or pruritus, which were similar to those reported in the trial setting. According to APASAL and AASLD guidelines [26,27], in patients with advanced liver fibrosis, HCV NS3/4A inhibitors, such as GLE/PIB, should be avoided due to the risk of liver decompensation during the treatment. Calculation of the CTP score is recommended to differentiate between compensated and decompensated cirrhosis. Therefore, careful review of the patient’s history of decompensation and documentation of having a Child–Pugh score <7 is required before initiating NS3/4A-containing therapy. Monitoring of patient liver function during the treatment is recommended for patients with advanced fibrosis. In our real-world practice, laboratory tests were performed at 2- to 4-week intervals. Two percent of the study population observed a transient elevation of liver function during the treatment course (Table 3). None of the patients discontinued therapy because of treatment-related adverse events, which indicates the safety of GLE/PIB in our real-world experience for patients with compensated cirrhosis, who fulfill the treatment criteria.

There are limited data available on the dynamic changes in liver stiffness among patients with cirrhosis who achieved SVR after interferon-free treatment. However, a significant decrease in the values of APRI and FIB-4 was observed among 164 patients with cirrhosis who achieved SVR with DAA [28]. The German Hepatitis C-Registry [29] found that patients with cirrhosis had a higher magnitude of transient elastography improvement between the baseline and follow-up (25.1 kPa vs. 21.5 kPa; *p* = 0.002) than those with F2–F3 (8.9 kPa vs. 8.8 kPa; *p* = 0.060) or F0–F1 (5.3 kPa vs. 5.2 kPa; *p* = 0.064). In a multicenter prospective study conducted in Italy, a long-term decline in liver stiffness was observed after achieving SVR, aside from the resolution of inflammation [30]. Fatty liver and the development of HCC interfered with the late reduction in liver stiffness. Patients with liver stiffness of ≥14 kPa at 12 weeks after the end of treatment had a higher risk of developing HCC.

The present study demonstrated a similar finding, with significant improvement in both FIB-4 (4.49 vs. 2.86, *p* < 0.001) and APRI scores (1.309 vs. 0.504, *p* < 0.001) at the baseline and after achieving SVR. Such an improvement, along with an improvement in liver function parameters, such as albumin level, bilirubin level, or prothrombin time, indicates that a beneficial effect can be achieved after SVR among patients with cirrhosis [31]. These patients may have a reduced risk of early HCC recurrence [32,33], cardiovascular risk [34], and progression towards diabetes risk [35].

The present study has several limitations. Firstly, it is a retrospective study, which means that there might be bias regarding side effects. The study included a relatively small number of patients, with an average age of 65 years, which does not permit the results of this study to be generalized to other age groups. Secondly, 75% of our patients were treatment-naïve, with either genotype 1 or 2. More experience is required to confirm the treatment results of GLE/PIB in patients with, for instance, less common genotypes, such as type 3 or 6. Thirdly, we did not assess the on-treatment HCV RNA test because the Taiwanese national health insurance system only reimburses the HCV RNA test for SVR and ETVR. We were not able to provide viral kinetic data, for further analysis, regarding the two patients who did not achieve SVR. Fourthly, our study only includes treatment-naïve patients who underwent DAA treatment. Therefore, we cannot extrapolate the efficacy data of GLE/PIB to patients who have previously undergone failed DAA therapy.

## 5. Conclusions

This study demonstrated that GLE/PIB-based therapy is highly effective in patients with compensated liver cirrhosis.

## Figures and Tables

**Table 1:** Baseline characteristics of the study population.

	All Patients (*n* = 90)	Eight-Week Course (*n* = 20)	≥Twelve-Week Course (*n* = 70)	*p*-Values
Gender, *n*/N (%)	52/90 (57.8%)	10/20 (50.0%)	42/70 (60.0%)	0.425
Age, y, mean ± SD	65 ± 12	69 ± 11	63 ± 12	0.104
HCC, *n*/N (%)	15/90 (16.7%)	7/20 (35.0%)	8/70 (11.4%)	0.036
Active HCC *, *n*/N (%)	6/15 (40%)	3/7 (42.9%)	3/8 (37.5%)	1.000
ESRD, *n*/N (%)	16/90 (17.8%)	2/20 (10.0%)	14/70 (20.0%)	0.508
PWID, *n*/N (%)	8/90 (8.9%)	0	8/70 (11.4%)	0.191
DM, *n*/N (%)	27/90 (30.0%)	6/20 (30.0%)	21/70 (30.0%)	1.000
HTN, *n*/N (%)	37/90 (41.1%)	6/20 (30.0%)	31/70 (44.3%)	0.252
HBV, *n*/N (%)	7/90 (7.8%)	3/20 (15.0%)	4/70 (5.7%)	0.181
HCV Genotype/N (%)				0.046
Type 1	36/90 (40.0%)	12/20 (60.0%)	24/70 (34.3%)	0.070
Type 2	32/90 (35.6%)	4/20 (20.0%)	28/70 (40.0%)	0.167
Type 3	12/90 (13.3%)	0/20 (0.0%)	12/70 (17.1%)	0.061
Type 6	3/90 (3.3%)	1/20 (5.0%)	2/70 (2.9%)	0.534
Unclassified type	7/90 (7.8%)	3/20 (15.0%)	4/70 (5.7%)	0.181
Prior intervention using interferon	11/90 (12.2%)	0	11/70 (15.7%)	0.114
Failure	9/90 (10.0%)	0	9/70 (12.9%)	
Interrupted	2/90 (2.2%)	0	2/70 (2.9%)	
HCV viral load, Log IU/mL, median (IQR)	5.85 (4.9–6.37)	5.85 (4.5–6.34)	5.85 (5.1–6.37)	0.522
GOT (AST), U/L, median (IQR)	54 (34–117)	91 (53–162)	45 (31–104)	0.004
GPT, U/L, median (IQR)	55 (33–138)	86 (43–185)	54 (30–133)	0.055
Platelet count, ×10³/μL, median (IQR)	125 (92–155)	137 (94–200)	120 (92–152)	0.277
Hb, g/dL, median (IQR)	13.1 (10.2–14)	13.1 (11.9–14)	13.1 (10.1–14)	0.512
Albumin, g/dL, mean ± SD	3.7 ± 0.42	3.75 ± 0.4	3.68 ± 0.43	0.552
Bilirubin-T, mg/dL, median (IQR)	0.7 (0.51–0.94)	0.68 (0.52–0.9)	0.71 (0.51–0.95)	0.473
Creatinine, mg/dL, median (IQR)	1.06 (0.8–2.63)	1.06 (0.72–1.96)	1.07 (0.8–4)	0.578
Prothrombin time (INR), mean ± SD	1.03 ± 0.08	1.04 ± 0.06	1.03 ± 0.09	0.746
APRI, median (IQR)	1.309 (0.664–2.847)	1.82 (1.107–3.517)	1.042 (0.63–2.629)	0.046
FIB4, median (IQR)	4.32 (2.39–7.41)	6.82 (3.75–7.36)	3.87 (2.36–7.41)	0.071

Abbreviations: n/N: case number of the disease/total case number; HCC: hepatocellular carcinoma; ESRD: end-stage renal disease; PWID: people who inject drugs; DM: diabetes mellitus; HTN: hypertension; HBV: hepatitis B; HCV: hepatitis C; GOT: glutamic oxaloacetic transaminase; GPT: glutamic pyruvic transaminase; Hb: hemoglobin; APRI: AST-to-platelet ratio index; FIB-4: fibrosis-4; * active HCC is defined as the presence of viable hepatocellular carcinoma at the initiation of antiviral therapy.

**Table 2:** Treatment response of the study population.

HCV RNA < LLOQ ^a^	All Patients (N = 90)		Eight-Week Course (N = 20)		≥Twelve-Week Course (N = 70)		*p*-Values
	*n*/N (%)	95% CI	*n*/N (%)	95% CI	*n*/N (%)	95% CI	
ETVR							
ITT	88/90 (97.8)	92.2–99.7	18/20 (90)	68.3–98.8	70/70 (100)	94.9–100	0.047
PP	88/89 (98.9)	93.9–100	18/19 (94.7)	73.9–99.9	70/70 (100)	94.9–100	0.213
Reason for non-ETVR, *n*							
Death ᵇ	1		1		0		0.222
SVR12							
ITT	85/90 (94.4)	87.5–98.2	17/20 (85)	62.1–96.8	68/70 (97.1)	90.0–99.6	0.071
PP	85/87 (97.7)	92.0–99.7	17/18 (94.4)	72.6–99.9	68/69 (98.6)	92.3–100	0.373
Reason for non-SVR12, *n*							
Death ^b^	1		1		0		0.222
Lost to follow-up	2		1		1		0.397

a. LLOQ, lower limit of qualification was 12 IU/mL. b. The patient passed away in their sleep. ITT: intention-to-treat; PP: per-protocol; ETVR: end-of-treatment virological response; SVR: sustained virological response; *n*/N: case number of the disease/total case number.

**Table 3:** Treatment side effects.

	All Patients	Eight-Week Course	≥Twelve-Week Course	*p*-Values
Clinical Side Effects				
Anorexia, *n*/N (%)	11/90 (12.2%)	2/20 (10.0%)	9/70 (12.9%)	1.000
Malaise, *n*/N (%)	7/90 (7.8%)	3/20 (15.0%)	4/70 (5.7%)	0.181
Abdominal discomfort, *n*/N (%)	7/90 (7.8%)	2/20 (10.0%)	5/70 (7.1%)	0.649
Pruritus, *n*/N (%)	7/90 (7.8%)	0	7/70 (10.0%)	0.341
Insomnia, *n*/N (%)	6/90 (6.7%)	1/20 (5.0%)	5/70 (7.1%)	1.000
Dizziness, *n*/N (%)	4/90 (4.4%)	1/20 (5.0%)	3/70 (4.3%)	1.000
GERD, *n*/N (%)	1/90 (1.1%)	1/20 (5.0%)	0	0.222
Laboratory Side Effects				
GOT, *n*/N (%)				0.016
<3× elevation	83/90 (92.2%)	16/20 (80.0%)	67/70 (95.7%)	
3–5× elevation	5/90 (5.6%)	2/20 (10.0%)	3/70 (4.3%)	
≥5× elevation	2/90 (2.2%)	2/20 (10.0%)	0	
GPT, *n*/N (%)				0.238
<3× elevation	84/90 (93.3%)	17/20 (85.0%)	67/70 (95.7%)	
3–5× elevation	4/90 (4.4%)	2/20 (10.0%)	2/70 (2.9%)	
≥5× elevation	2/90 (2.2%)	1/20 (5.0%)	1/70 (1.4%)	
Bilirubin, *n*/N (%)				0.437
<1.5× elevation	75/90 (83.3%)	15/20 (75.0%)	60/70 (85.7%)	
1.5–3× elevation	13/90 (14.4%)	4/20 (20.0%)	9/70 (12.9%)	
≥3×elevation	2/90 (2.2%)	1/20 (5.0%)	1/70 (1.4%)	
CTCAE Hemoglobin, *n*/N (%)				0.367
G0	28/79 (35.4%)	7/17 (41.2%)	21/62 (33.9%)	
G1	29/79 (36.7%)	8/17 (47.1%)	21/62 (33.9%)	
G2	17/79 (21.5%)	2/17 (11.8%)	15/62 (24.2%)	
G3	5/79 (6.3%)	0	5/62 (8.1%)	
CTCAE Thrombocytopenia, *n*/N (%)				0.303
G0	25/78 (32.1%)	8/17 (47.1%)	17/61 (27.9%)	
G1	38/78 (48.7%)	6/17 (35.3%)	32/61 (52.5%)	
G2	11/78 (14.1%)	3/17 (17.6%)	8/61 (13.1%)	
G3	4/78 (5.1%)	0	4/61 (6.6%)	

**Table 4:** Change in laboratory parameters after achieving SVR12.

Item	Baseline	After SVR12	*p*-Value
GOT (AST), U/median (IQR)	55 (36–117)	25 (20–31)	<0.001
GPT, U/L, median (IQR)	55 (33–138)	20 (14–28)	<0.001
Platelet count, ×10³/μL, median (IQR)	120 (90–152)	122 (94–177)	0.219
Hb, g/dL, median (IQR)	12.9 (10.2–13.9)	13 (10.3–14.3)	0.404
Prothrombin time (INR), median (IQR)	1.03 (0.98–1.08)	1.02 (0.97–1.06)	0.011
Albumin, g/dL, median (IQR)	3.7 (3.5–4)	3.9 (3.7–4.2)	<0.001
Bilirubin-T, mg/dL, median (IQR)	0.7 (0.51–0.94)	0.6 (0.45–0.8)	0.005
Creatinine, mg/dL, median (IQR)	1.07 (0.79–2.66)	1.03 (0.81–2.77)	0.464
FIB4, median (IQR)	4.49 (2.39–7.44)	2.86 (1.84–4.6)	<0.001
APRI, median (IQR)	1.309 (0.665–2.853)	0.504 (0.317–0.826)	<0.001
MELD score, median (IQR)	6.905 (4.31–13.457)	5.47 (3.065–13.81)	0.012

Abbreviations: GOT: glutamic oxaloacetic transaminase; GPT: glutamic pyruvic transaminase; Hb: hemoglobin; APRI: AST-to-platelet ratio index; FIB-4: fibrosis-4; MELD: model of end-stage liver disease.
